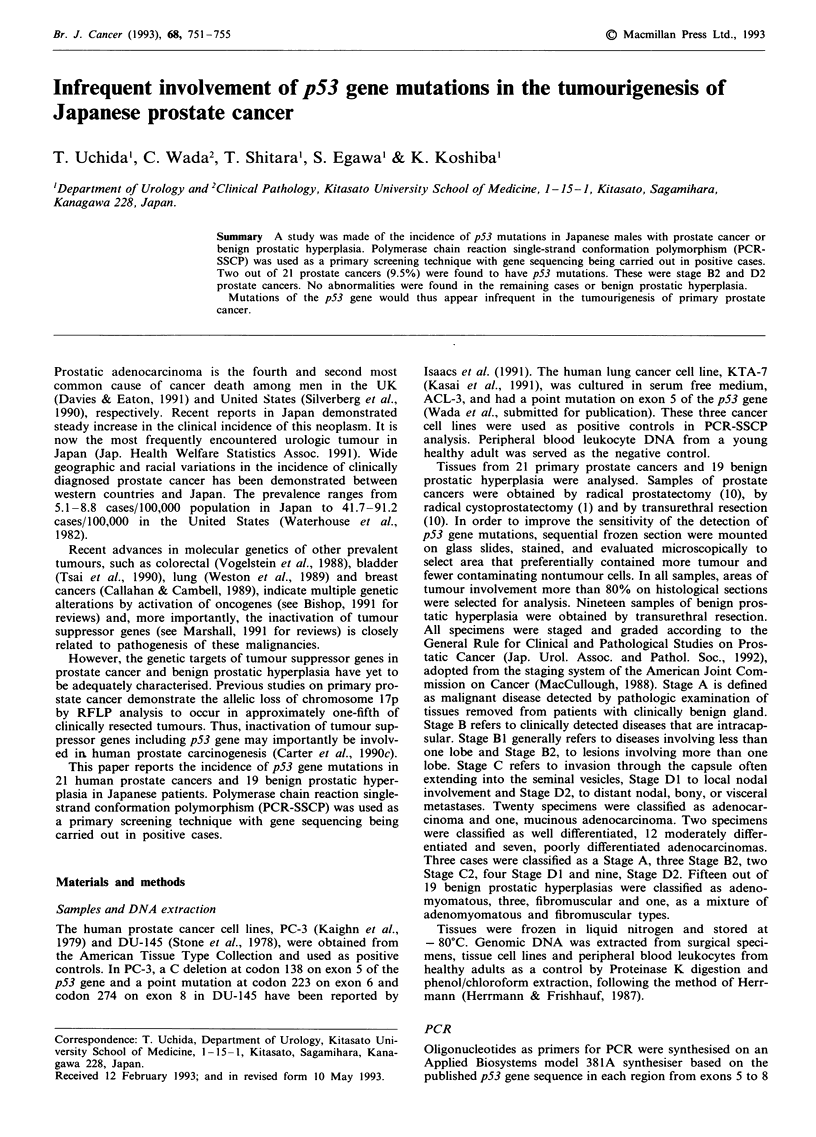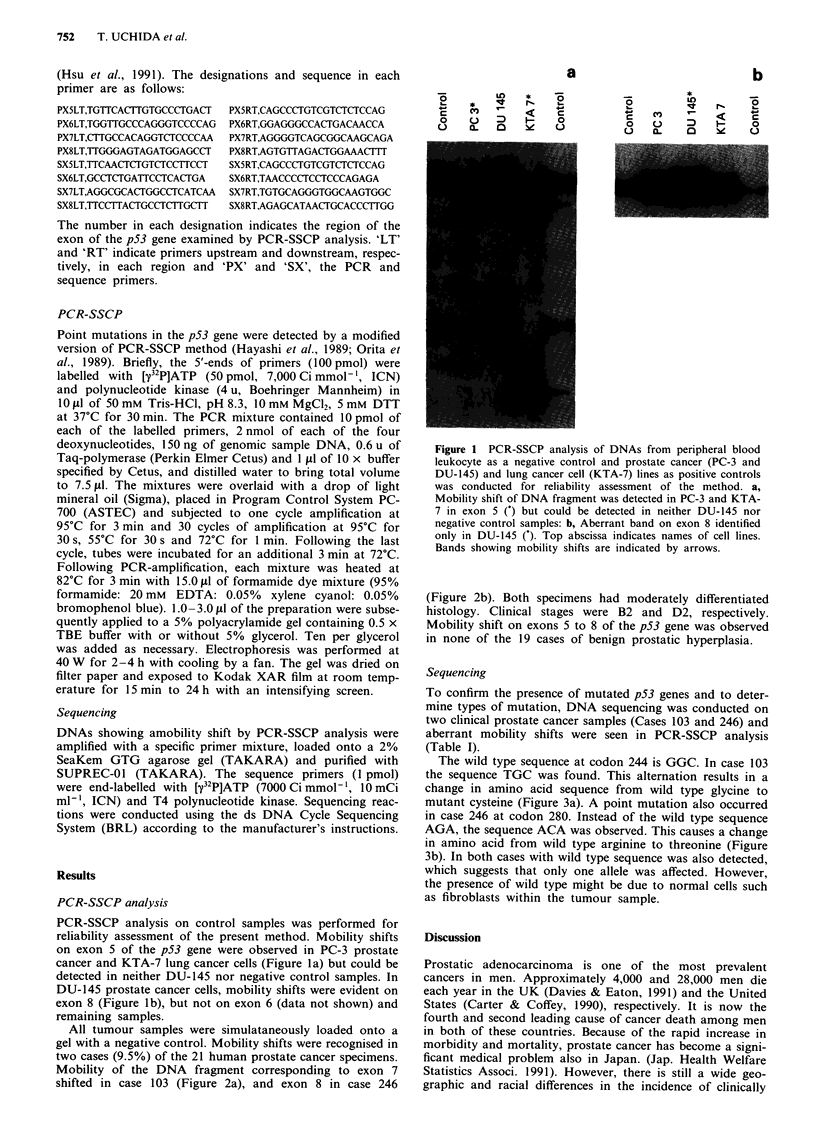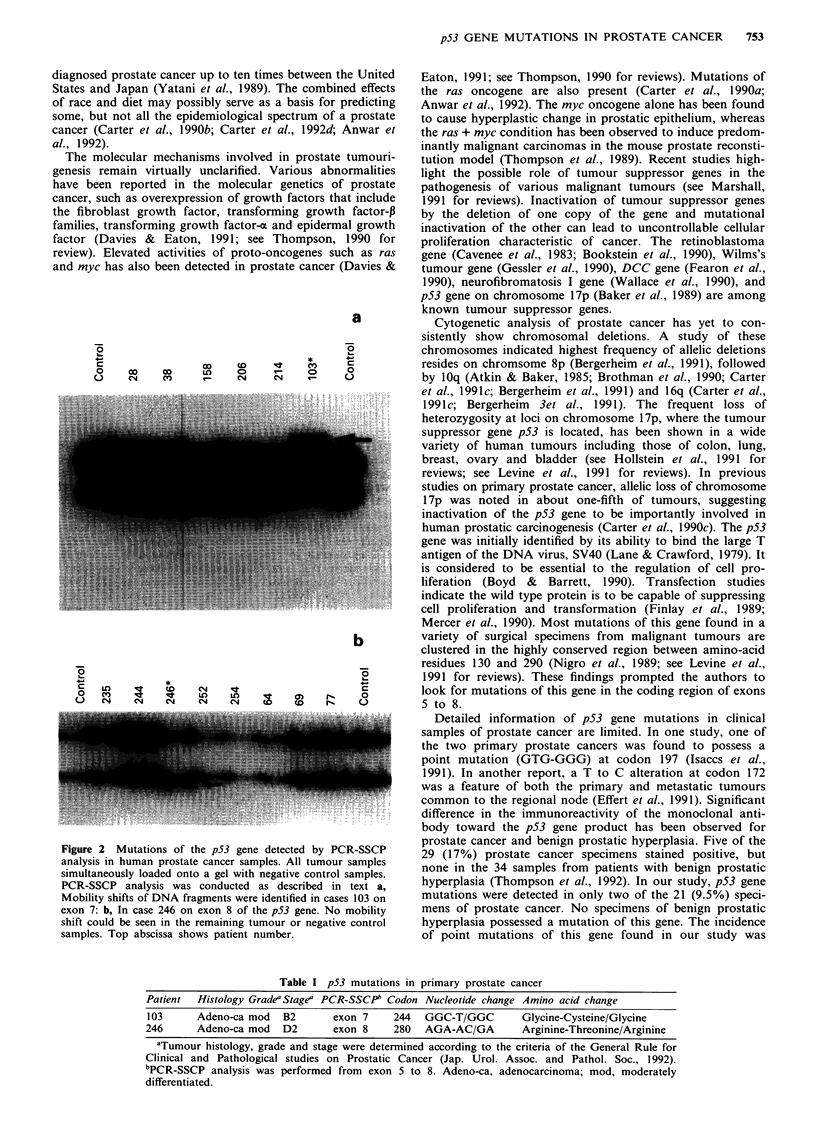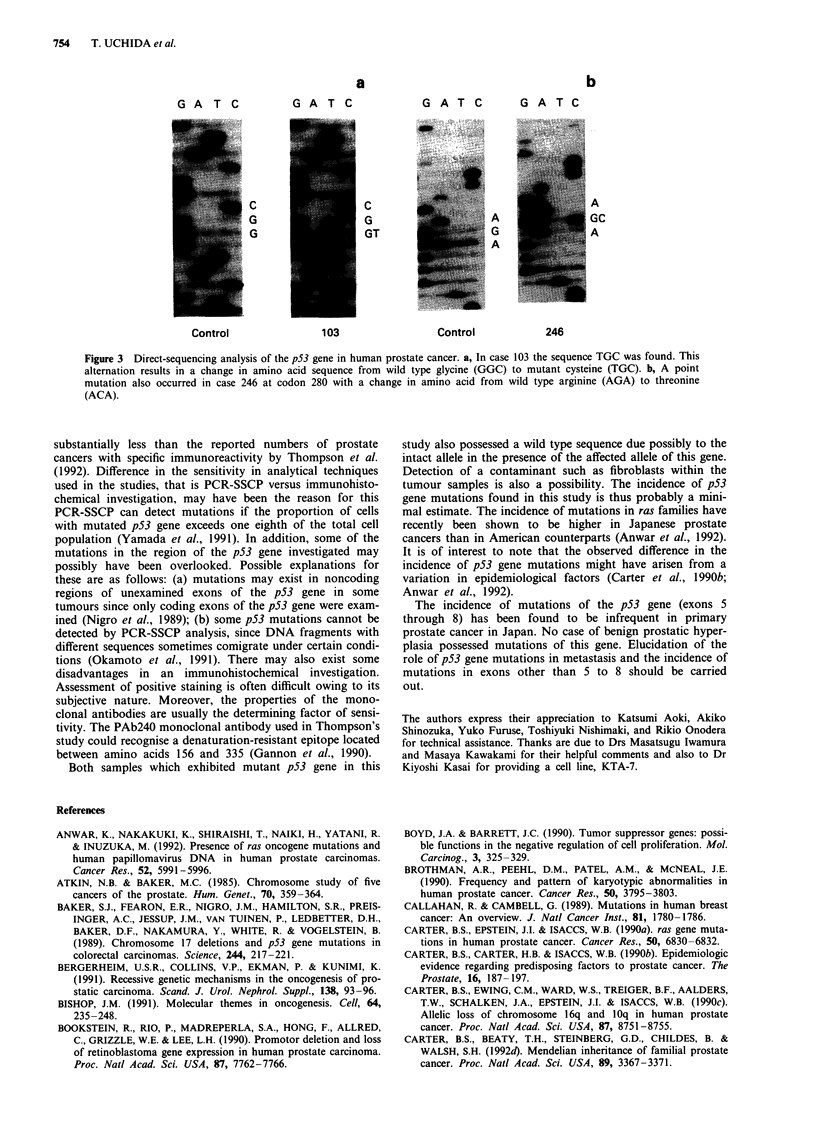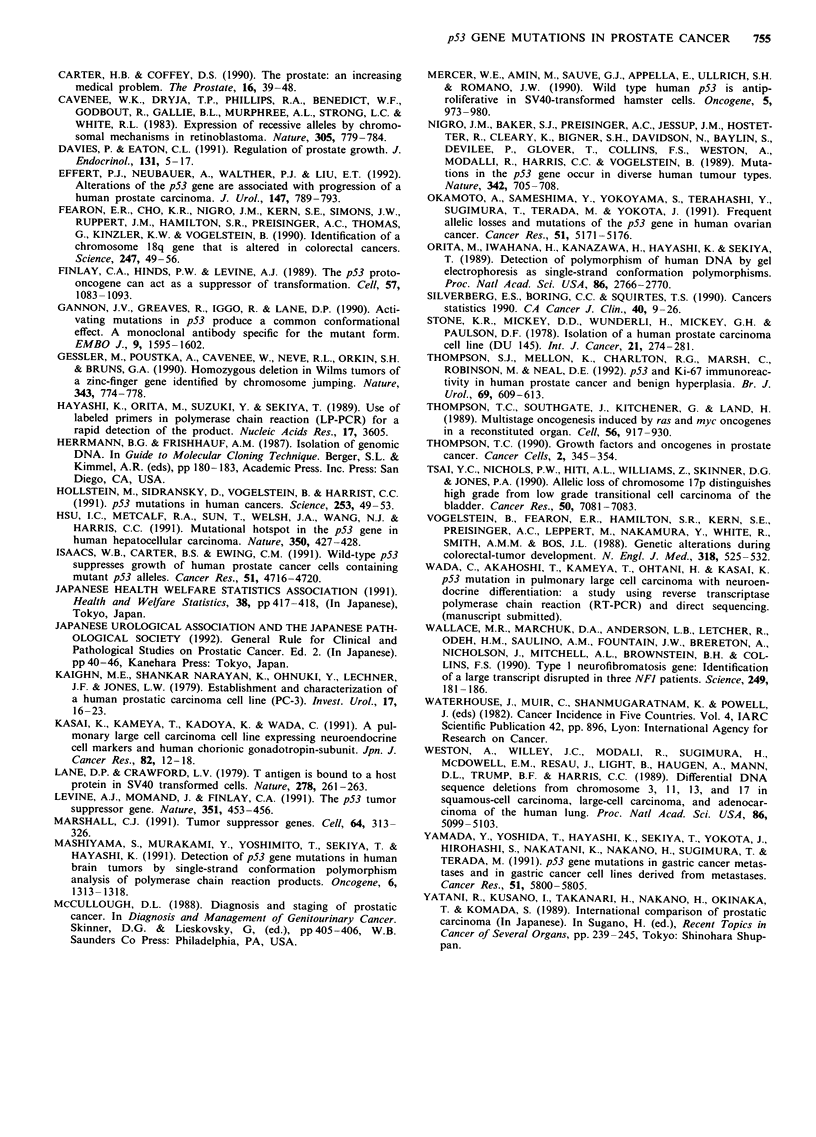# Infrequent involvement of p53 gene mutations in the tumourigenesis of Japanese prostate cancer.

**DOI:** 10.1038/bjc.1993.423

**Published:** 1993-10

**Authors:** T. Uchida, C. Wada, T. Shitara, S. Egawa, K. Koshiba

**Affiliations:** Department of Urology, Kitasato University School of Medicine, Kanagawa, Japan.

## Abstract

**Images:**


Br. J. Cancer (1993), 68, 751-755 © Macmillan Press Ltd., 1993

### Infrequent involvement of *p53* gene mutations in the tumourigenesis of Japanese prostate cancer

T. Uchida[1], C. Wada[2], T. Shitara[1], S. Egawa[1] & K. Koshiba[1]

[1]*Department of Urology and* [2]*Clinical Pathology, Kitasato University School of Medicine, 1-15-1, Kitasato, Sagamihara, Kanagawa 228, Japan.*

**Summary** A study was made of the incidence of *p53* mutations in Japanese males with prostate cancer or benign prostatic hyperplasia. Polymerase chain reaction single-strand conformation polymorphism (PCR-SSCP) was used as a primary screening technique with gene sequencing being carried out in positive cases. Two out of 21 prostate cancers (9.5%) were found to have *p53* mutations. These were stage B2 and D2 prostate cancers. No abnormalities were found in the remaining cases or benign prostatic hyperplasia.

Mutations of the *p53* gene would thus appear infrequent in the tumourigenesis of primary prostate cancer.

Prostatic adenocarcinoma is the fourth and second most common cause of cancer death among men in the UK (Davies & Eaton, 1991) and United States (Silverberg *et al.*, 1990), respectively. Recent reports in Japan demonstrated steady increase in the clinical incidence of this neoplasm. It is now the most frequently encountered urologic tumour in Japan (Jap. Health Welfare Statistics Assoc. 1991). Wide geographic and racial variations in the incidence of clinically diagnosed prostate cancer has been demonstrated between western countries and Japan. The prevalence ranges from 5.1-8.8 cases/100,000 population in Japan to 41.7-91.2 cases/100,000 in the United States (Waterhouse *et al.*, 1982).

Recent advances in molecular genetics of other prevalent tumours, such as colorectal (Vogelstein *et al.*, 1988), bladder (Tsai *et al.*, 1990), lung (Weston *et al.*, 1989) and breast cancers (Callahan & Cambell, 1989), indicate multiple genetic alterations by activation of oncogenes (see Bishop, 1991 for reviews) and, more importantly, the inactivation of tumour suppressor genes (see Marshall, 1991 for reviews) is closely related to pathogenesis of these malignancies.

However, the genetic targets of tumour suppressor genes in prostate cancer and benign prostatic hyperplasia have yet to be adequately characterised. Previous studies on primary prostate cancer demonstrate the allelic loss of chromosome 17p by RFLP analysis to occur in approximately one-fifth of clinically resected tumours. Thus, inactivation of tumour suppressor genes including *p53* gene may importantly be involved in human prostate carcinogenesis (Carter *et al.*, 1990*c*).

This paper reports the incidence of *p53* gene mutations in 21 human prostate cancers and 19 benign prostatic hyperplasia in Japanese patients. Polymerase chain reaction single-strand conformation polymorphism (PCR-SSCP) was used as a primary screening technique with gene sequencing being carried out in positive cases.

### Materials and methods

### *Samples and DNA extraction*

The human prostate cancer cell lines, PC-3 (Kaighn *et al.*, 1979) and DU-145 (Stone *et al.*, 1978), were obtained from the American Tissue Type Collection and used as positive controls. In PC-3, a C deletion at codon 138 on exon 5 of the *p53* gene and a point mutation at codon 223 on exon 6 and codon 274 on exon 8 in DU-145 have been reported by Isaacs *et al.* (1991). The human lung cancer cell line, KTA-7 (Kasai *et al.*, 1991), was cultured in serum free medium, ACL-3, and had a point mutation on exon 5 of the *p53* gene (Wada *et al.*, submitted for publication). These three cancer cell lines were used as positive controls in PCR-SSCP analysis. Peripheral blood leukocyte DNA from a young healthy adult was served as the negative control.

Tissues from 21 primary prostate cancers and 19 benign prostatic hyperplasia were analysed. Samples of prostate cancers were obtained by radical prostatectomy (10), by radical cystoprostatectomy (1) and by transurethral resection (10). In order to improve the sensitivity of the detection of *p53* gene mutations, sequential frozen section were mounted on glass slides, stained, and evaluated microscopically to select area that preferentially contained more tumour and fewer contaminating nontumour cells. In all samples, areas of tumour involvement more than 80% on histological sections were selected for analysis. Nineteen samples of benign prostatic hyperplasia were obtained by transurethral resection. All specimens were staged and graded according to the General Rule for Clinical and Pathological Studies on Prostatic Cancer (Jap. Urol. Assoc. and Pathol. Soc., 1992), adopted from the staging system of the American Joint Commission on Cancer (MacCullough, 1988). Stage A is defined as malignant disease detected by pathologic examination of tissues removed from patients with clinically benign gland. Stage B refers to clinically detected diseases that are intracapsular. Stage B1 generally refers to diseases involving less than one lobe and Stage B2, to lesions involving more than one lobe. Stage C refers to invasion through the capsule often extending into the seminal vesicles, Stage D1 to local nodal involvement and Stage D2, to distant nodal, bony, or visceral metastases. Twenty specimens were classified as adenocarcinoma and one, mucinous adenocarcinoma. Two specimens were classified as well differentiated, 12 moderately differentiated and seven, poorly differentiated adenocarcinomas. Three cases were classified as a Stage A, three Stage B2, two Stage C2, four Stage D1 and nine, Stage D2. Fifteen out of 19 benign prostatic hyperplasias were classified as adenomyomatous, three, fibromuscular and one, as a mixture of adenomyomatous and fibromuscular types.

Tissues were frozen in liquid nitrogen and stored at − 80°C. Genomic DNA was extracted from surgical specimens, tissue cell lines and peripheral blood leukocytes from healthy adults as a control by Proteinase K digestion and phenol/chloroform extraction, following the method of Herrmann (Herrmann & Frishhauf, 1987).

### *PCR*

Oligonucleotides as primers for PCR were synthesised on an Applied Biosystems model 381A synthesiser based on the published *p53* gene sequence in each region from exons 5 to 8

Correspondence: T. Uchida, Department of Urology, Kitasato University School of Medicine, 1-15-1, Kitasato, Sagamihara, Kanagawa 228, Japan.
Received 12 February 1993; and in revised form 10 May 1993.

(Hsu *et al.*, 1991). The designations and sequence in each primer are as follows:

| | |
|---|---|
| PX5LT,TGTTCACTTGTGCCCTGACT | PX5RT,CAGCCCTGTCGTCTCTCCAG |
| PX6LT,TGGTTGCCCAGGGTCCCCAG | PX6RT,GGAGGGCCACTGACAACCA |
| PX7LT,CTTGCCACAGGTCTCCCCAA | PX7RT,AGGGGTCAGCGGCAAGCAGA |
| PX8LT,TTGGGAGTAGATGGAGCCT | PX8RT,AGTGTTAGACTGGAAACTTT |
| SX5LT,TTCAACTCTGTCTCCTTCCT | SX5RT,CAGCCCTGTCGTCTCTCCAG |
| SX6LT,GCCTCTGATTCCTCACTGA | SX6RT,TAACCCCTCCTCCCAGAGA |
| SX7LT,AGGCGCACTGGCCTCATCAA | SX7RT,TGTGCAGGGTGGCAAGTGGC |
| SX8LT,TTCCTTACTGCCTCTTGCTT | SX8RT,AGAGCATAACTGCACCCTTGG |

The number in each designation indicates the region of the exon of the *p53* gene examined by PCR-SSCP analysis. 'LT' and 'RT' indicate primers upstream and downstream, respectively, in each region and 'PX' and 'SX', the PCR and sequence primers.

### *PCR-SSCP*

Point mutations in the *p53* gene were detected by a modified version of PCR-SSCP method (Hayashi *et al.*, 1989; Orita *et al.*, 1989). Briefly, the 5′-ends of primers (100 pmol) were labelled with [$\gamma^{32}$P]ATP (50 pmol, 7,000 Ci mmol$^{-1}$, ICN) and polynucleotide kinase (4 u, Boehringer Mannheim) in 10 µl of 50 mM Tris-HCl, pH 8.3, 10 mM $MgCl_2$, 5 mM DTT at 37°C for 30 min. The PCR mixture contained 10 pmol of each of the labelled primers, 2 nmol of each of the four deoxynucleotides, 150 ng of genomic sample DNA, 0.6 u of Taq-polymerase (Perkin Elmer Cetus) and 1 µl of 10 × buffer specified by Cetus, and distilled water to bring total volume to 7.5 µl. The mixtures were overlaid with a drop of light mineral oil (Sigma), placed in Program Control System PC-700 (ASTEC) and subjected to one cycle amplification at 95°C for 3 min and 30 cycles of amplification at 95°C for 30 s, 55°C for 30 s and 72°C for 1 min. Following the last cycle, tubes were incubated for an additional 3 min at 72°C. Following PCR-amplification, each mixture was heated at 82°C for 3 min with 15.0 µl of formamide dye mixture (95% formamide: 20 mM EDTA: 0.05% xylene cyanol: 0.05% bromophenol blue). 1.0–3.0 µl of the preparation were subsequently applied to a 5% polyacrylamide gel containing 0.5 × TBE buffer with or without 5% glycerol. Ten per glycerol was added as necessary. Electrophoresis was performed at 40 W for 2–4 h with cooling by a fan. The gel was dried on filter paper and exposed to Kodak XAR film at room temperature for 15 min to 24 h with an intensifying screen.

### *Sequencing*

DNAs showing amobility shift by PCR-SSCP analysis were amplified with a specific primer mixture, loaded onto a 2% SeaKem GTG agarose gel (TAKARA) and purified with SUPREC-01 (TAKARA). The sequence primers (1 pmol) were end-labelled with [$\gamma^{32}$P]ATP (7000 Ci mmol$^{-1}$, 10 mCi ml$^{-1}$, ICN) and T4 polynucleotide kinase. Sequencing reactions were conducted using the ds DNA Cycle Sequencing System (BRL) according to the manufacturer's instructions.

### Results

### *PCR-SSCP analysis*

PCR-SSCP analysis on control samples was performed for reliability assessment of the present method. Mobility shifts on exon 5 of the *p53* gene were observed in PC-3 prostate cancer and KTA-7 lung cancer cells (Figure 1a) but could be detected in neither DU-145 nor negative control samples. In DU-145 prostate cancer cells, mobility shifts were evident on exon 8 (Figure 1b), but not on exon 6 (data not shown) and remaining samples.

All tumour samples were simulataneously loaded onto a gel with a negative control. Mobility shifts were recognised in two cases (9.5%) of the 21 human prostate cancer specimens. Mobility of the DNA fragment corresponding to exon 7 shifted in case 103 (Figure 2a), and exon 8 in case 246 (Figure 2b). Both specimens had moderately differentiated histology. Clinical stages were B2 and D2, respectively. Mobility shift on exons 5 to 8 of the *p53* gene was observed in none of the 19 cases of benign prostatic hyperplasia.

**Figure 1** PCR-SSCP analysis of DNAs from peripheral blood leukocyte as a negative control and prostate cancer (PC-3 and DU-145) and lung cancer cell (KTA-7) lines as positive controls was conducted for reliability assessment of the method. **a,** Mobility shift of DNA fragment was detected in PC-3 and KTA-7 in exon 5 (*) but could be detected in neither DU-145 nor negative control samples: **b,** Aberrant band on exon 8 identified only in DU-145 (*). Top abscissa indicates names of cell lines. Bands showing mobility shifts are indicated by arrows.

### *Sequencing*

To confirm the presence of mutated *p53* genes and to determine types of mutation, DNA sequencing was conducted on two clinical prostate cancer samples (Cases 103 and 246) and aberrant mobility shifts were seen in PCR-SSCP analysis (Table I).

The wild type sequence at codon 244 is GGC. In case 103 the sequence TGC was found. This alternation results in a change in amino acid sequence from wild type glycine to mutant cysteine (Figure 3a). A point mutation also occurred in case 246 at codon 280. Instead of the wild type sequence AGA, the sequence ACA was observed. This causes a change in amino acid from wild type arginine to threonine (Figure 3b). In both cases with wild type sequence was also detected, which suggests that only one allele was affected. However, the presence of wild type might be due to normal cells such as fibroblasts within the tumour sample.

### Discussion

Prostatic adenocarcinoma is one of the most prevalent cancers in men. Approximately 4,000 and 28,000 men die each year in the UK (Davies & Eaton, 1991) and the United States (Carter & Coffey, 1990), respectively. It is now the fourth and second leading cause of cancer death among men in both of these countries. Because of the rapid increase in morbidity and mortality, prostate cancer has become a significant medical problem also in Japan. (Jap. Health Welfare Statistics Associ. 1991). However, there is still a wide geographic and racial differences in the incidence of clinically diagnosed prostate cancer up to ten times between the United States and Japan (Yatani *et al.*, 1989). The combined effects of race and diet may possibly serve as a basis for predicting some, but not all the epidemiological spectrum of a prostate cancer (Carter *et al.*, 1990*b*; Carter *et al.*, 1992*d*; Anwar *et al.*, 1992).

The molecular mechanisms involved in prostate tumourigenesis remain virtually unclarified. Various abnormalities have been reported in the molecular genetics of prostate cancer, such as overexpression of growth factors that include the fibroblast growth factor, transforming growth factor-β families, transforming growth factor-α and epidermal growth factor (Davies & Eaton, 1991; see Thompson, 1990 for review). Elevated activities of proto-oncogenes such as *ras* and *myc* has also been detected in prostate cancer (Davies & Eaton, 1991; see Thompson, 1990 for reviews). Mutations of the *ras* oncogene are also present (Carter *et al.*, 1990*a*; Anwar *et al.*, 1992). The *myc* oncogene alone has been found to cause hyperplastic change in prostatic epithelium, whereas the *ras* + *myc* condition has been observed to induce predominantly malignant carcinomas in the mouse prostate reconstitution model (Thompson *et al.*, 1989). Recent studies highlight the possible role of tumour suppressor genes in the pathogenesis of various malignant tumours (see Marshall, 1991 for reviews). Inactivation of tumour suppressor genes by the deletion of one copy of the gene and mutational inactivation of the other can lead to uncontrollable cellular proliferation characteristic of cancer. The retinoblastoma gene (Cavenee *et al.*, 1983; Bookstein *et al.*, 1990), Wilms's tumour gene (Gessler *et al.*, 1990), *DCC* gene (Fearon *et al.*, 1990), neurofibromatosis I gene (Wallace *et al.*, 1990), and *p53* gene on chromosome 17p (Baker *et al.*, 1989) are among known tumour suppressor genes.

**Figure 2** Mutations of the *p53* gene detected by PCR-SSCP analysis in human prostate cancer samples. All tumour samples simultaneously loaded onto a gel with negative control samples. PCR-SSCP analysis was conducted as described in text **a**, Mobility shifts of DNA fragments were identified in cases 103 on exon 7: **b**, In case 246 on exon 8 of the *p53* gene. No mobility shift could be seen in the remaining tumour or negative control samples. Top abscissa shows patient number.

Cytogenetic analysis of prostate cancer has yet to consistently show chromosomal deletions. A study of these chromosomes indicated highest frequency of allelic deletions resides on chromsome 8p (Bergerheim *et al.*, 1991), followed by 10q (Atkin & Baker, 1985; Brothman *et al.*, 1990; Carter *et al.*, 1991*c*; Bergerheim *et al.*, 1991) and 16q (Carter *et al.*, 1991*c*; Bergerheim 3*et al.*, 1991). The frequent loss of heterozygosity at loci on chromosome 17p, where the tumour suppressor gene *p53* is located, has been shown in a wide variety of human tumours including those of colon, lung, breast, ovary and bladder (see Hollstein *et al.*, 1991 for reviews; see Levine *et al.*, 1991 for reviews). In previous studies on primary prostate cancer, allelic loss of chromosome 17p was noted in about one-fifth of tumours, suggesting inactivation of the *p53* gene to be importantly involved in human prostatic carcinogenesis (Carter *et al.*, 1990*c*). The *p53* gene was initially identified by its ability to bind the large T antigen of the DNA virus, SV40 (Lane & Crawford, 1979). It is considered to be essential to the regulation of cell proliferation (Boyd & Barrett, 1990). Transfection studies indicate the wild type protein is to be capable of suppressing cell proliferation and transformation (Finlay *et al.*, 1989; Mercer *et al.*, 1990). Most mutations of this gene found in a variety of surgical specimens from malignant tumours are clustered in the highly conserved region between amino-acid residues 130 and 290 (Nigro *et al.*, 1989; see Levine *et al.*, 1991 for reviews). These findings prompted the authors to look for mutations of this gene in the coding region of exons 5 to 8.

Detailed information of *p53* gene mutations in clinical samples of prostate cancer are limited. In one study, one of the two primary prostate cancers was found to possess a point mutation (GTG-GGG) at codon 197 (Isaccs *et al.*, 1991). In another report, a T to C alteration at codon 172 was a feature of both the primary and metastatic tumours common to the regional node (Effert *et al.*, 1991). Significant difference in the immunoreactivity of the monoclonal antibody toward the *p53* gene product has been observed for prostate cancer and benign prostatic hyperplasia. Five of the 29 (17%) prostate cancer specimens stained positive, but none in the 34 samples from patients with benign prostatic hyperplasia (Thompson *et al.*, 1992). In our study, *p53* gene mutations were detected in only two of the 21 (9.5%) specimens of prostate cancer. No specimens of benign prostatic hyperplasia possessed a mutation of this gene. The incidence of point mutations of this gene found in our study was

**Table I** *p53* mutations in primary prostate cancer

| *Patient* | *Histology Grade*[a] | *Stage*[a] | *PCR-SSCP*[b] | *Codon* | *Nucleotide change* | *Amino acid change* |
|---|---|---|---|---|---|---|
| 103 | Adeno-ca mod | B2 | exon 7 | 244 | GGC-T/GGC | Glycine-Cysteine/Glycine |
| 246 | Adeno-ca mod | D2 | exon 8 | 280 | AGA-AC/GA | Arginine-Threonine/Arginine |

[a]Tumour histology, grade and stage were determined according to the criteria of the General Rule for Clinical and Pathological studies on Prostatic Cancer (Jap. Urol. Assoc. and Pathol. Soc., 1992). [b]PCR-SSCP analysis was performed from exon 5 to 8. Adeno-ca, adenocarcinoma; mod, moderately differentiated.

**Figure 3** Direct-sequencing analysis of the *p53* gene in human prostate cancer. **a**, In case 103 the sequence TGC was found. This alternation results in a change in amino acid sequence from wild type glycine (GGC) to mutant cysteine (TGC). **b**, A point mutation also occurred in case 246 at codon 280 with a change in amino acid from wild type arginine (AGA) to threonine (ACA).

substantially less than the reported numbers of prostate cancers with specific immunoreactivity by Thompson *et al.* (1992). Difference in the sensitivity in analytical techniques used in the studies, that is PCR-SSCP versus immunohistochemical investigation, may have been the reason for this PCR-SSCP can detect mutations if the proportion of cells with mutated *p53* gene exceeds one eighth of the total cell population (Yamada *et al.*, 1991). In addition, some of the mutations in the region of the *p53* gene investigated may possibly have been overlooked. Possible explanations for these are as follows: (a) mutations may exist in noncoding regions of unexamined exons of the *p53* gene in some tumours since only coding exons of the *p53* gene were examined (Nigro *et al.*, 1989); (b) some *p53* mutations cannot be detected by PCR-SSCP analysis, since DNA fragments with different sequences sometimes comigrate under certain conditions (Okamoto *et al.*, 1991). There may also exist some disadvantages in an immunohistochemical investigation. Assessment of positive staining is often difficult owing to its subjective nature. Moreover, the properties of the monoclonal antibodies are usually the determining factor of sensitivity. The PAb240 monoclonal antibody used in Thompson's study could recognise a denaturation-resistant epitope located between amino acids 156 and 335 (Gannon *et al.*, 1990).

Both samples which exhibited mutant *p53* gene in this study also possessed a wild type sequence due possibly to the intact allele in the presence of the affected allele of this gene. Detection of a contaminant such as fibroblasts within the tumour samples is also a possibility. The incidence of *p53* gene mutations found in this study is thus probably a minimal estimate. The incidence of mutations in *ras* families have recently been shown to be higher in Japanese prostate cancers than in American counterparts (Anwar *et al.*, 1992). It is of interest to note that the observed difference in the incidence of *p53* gene mutations might have arisen from a variation in epidemiological factors (Carter *et al.*, 1990*b*; Anwar *et al.*, 1992).

The incidence of mutations of the *p53* gene (exons 5 through 8) has been found to be infrequent in primary prostate cancer in Japan. No case of benign prostatic hyperplasia possessed mutations of this gene. Elucidation of the role of *p53* gene mutations in metastasis and the incidence of mutations in exons other than 5 to 8 should be carried out.

The authors express their appreciation to Katsumi Aoki, Akiko Shinozuka, Yuko Furuse, Toshiyuki Nishimaki, and Rikio Onodera for technical assistance. Thanks are due to Drs Masatsugu Iwamura and Masaya Kawakami for their helpful comments and also to Dr Kiyoshi Kasai for providing a cell line, KTA-7.

### References

ANWAR, K., NAKAKUKI, K., SHIRAISHI, T., NAIKI, H., YATANI, R. & INUZUKA, M. (1992). Presence of *ras* oncogene mutations and human papillomavirus DNA in human prostate carcinomas. *Cancer Res.*, **52**, 5991-5996.

ATKIN, N.B. & BAKER, M.C. (1985). Chromosome study of five cancers of the prostate. *Hum. Genet.*, **70**, 359-364.

BAKER, S.J., FEARON, E.R., NIGRO, J.M., HAMILTON, S.R., PREISINGER, A.C., JESSUP, J.M., VAN TUINEN, P., LEDBETTER, D.H., BAKER, D.F., NAKAMURA, Y., WHITE, R. & VOGELSTEIN, B. (1989). Chromosome 17 deletions and *p53* gene mutations in colorectal carcinomas. *Science*, **244**, 217-221.

BERGERHEIM, U.S.R., COLLINS, V.P., EKMAN, P. & KUNIMI, K. (1991). Recessive genetic mechanisms in the oncogenesis of prostatic carcinoma. *Scand. J. Urol. Nephrol. Suppl.*, **138**, 93-96.

BISHOP, J.M. (1991). Molecular themes in oncogenesis. *Cell*, **64**, 235-248.

BOOKSTEIN, R., RIO, P., MADREPERLA, S.A., HONG, F., ALLRED, C., GRIZZLE, W.E. & LEE, L.H. (1990). Promotor deletion and loss of retinoblastoma gene expression in human prostate carcinoma. *Proc. Natl Acad. Sci. USA*, **87**, 7762-7766.

BOYD, J.A. & BARRETT, J.C. (1990). Tumor suppressor genes: possible functions in the negative regulation of cell proliferation. *Mol. Carcinog.*, **3**, 325-329.

BROTHMAN, A.R., PEEHL, D.M., PATEL, A.M., & MCNEAL, J.E. (1990). Frequency and pattern of karyotypic abnormalities in human prostate cancer. *Cancer Res.*, **50**, 3795-3803.

CALLAHAN, R. & CAMBELL, G. (1989). Mutations in human breast cancer: An overview. *J. Natl Cancer Inst.*, **81**, 1780-1786.

CARTER, B.S., EPSTEIN, J.I. & ISACCS, W.B. (1990*a*). *ras* gene mutations in human prostate cancer. *Cancer Res.*, **50**, 6830-6832.

CARTER, B.S., CARTER, H.B. & ISACCS, W.B. (1990*b*). Epidemiologic evidence regarding predisposing factors to prostate cancer. *The Prostate*, **16**, 187-197.

CARTER, B.S., EWING, C.M., WARD, W.S., TREIGER, B.F., AALDERS, T.W., SCHALKEN, J.A., EPSTEIN, J.I. & ISACCS, W.B. (1990*c*). Allelic loss of chromosome 16q and 10q in human prostate cancer. *Proc. Natl Acad. Sci. USA*, **87**, 8751-8755.

CARTER, B.S., BEATY, T.H., STEINBERG, G.D., CHILDES, B. & WALSH, S.H. (1992*d*). Mendelian inheritance of familial prostate cancer. *Proc. Natl Acad. Sci. USA*, **89**, 3367-3371.

CARTER, H.B. & COFFEY, D.S. (1990). The prostate: an increasing medical problem. *The Prostate*, **16**, 39–48.

CAVENEE, W.K., DRYJA, T.P., PHILLIPS, R.A., BENEDICT, W.F., GODBOUT, R., GALLIE, B.L., MURPHREE, A.L., STRONG, L.C. & WHITE, R.L. (1983). Expression of recessive alleles by chromosomal mechanisms in retinoblastoma. *Nature*, **305**, 779–784.

DAVIES, P. & EATON, C.L. (1991). Regulation of prostate growth. *J. Endocrinol.*, **131**, 5–17.

EFFERT, P.J., NEUBAUER, A., WALTHER, P.J. & LIU, E.T. (1992). Alterations of the *p53* gene are associated with progression of a human prostate carcinoma. *J. Urol.*, **147**, 789–793.

FEARON, E.R., CHO, K.R., NIGRO, J.M., KERN, S.E., SIMONS, J.W., RUPPERT, J.M., HAMILTON, S.R., PREISINGER, A.C., THOMAS, G., KINZLER, K.W. & VOGELSTEIN, B. (1990). Identification of a chromosome 18q gene that is altered in colorectal cancers. *Science*, **247**, 49–56.

FINLAY, C.A., HINDS, P.W. & LEVINE, A.J. (1989). The *p53* protooncogene can act as a suppressor of transformation. *Cell*, **57**, 1083–1093.

GANNON, J.V., GREAVES, R., IGGO, R. & LANE, D.P. (1990). Activating mutations in *p53* produce a common conformational effect. A monoclonal antibody specific for the mutant form. *EMBO J.*, **9**, 1595–1602.

GESSLER, M., POUSTKA, A., CAVENEE, W., NEVE, R.L., ORKIN, S.H. & BRUNS, G.A. (1990). Homozygous deletion in Wilms tumors of a zinc-finger gene identified by chromosome jumping. *Nature*, **343**, 774–778.

HAYASHI, K., ORITA, M., SUZUKI, Y. & SEKIYA, T. (1989). Use of labeled primers in polymerase chain reaction (LP-PCR) for a rapid detection of the product. *Nucleic Acids Res.*, **17**, 3605.

HERRMANN, B.G. & FRISHHAUF, A.M. (1987). Isolation of genomic DNA. In *Guide to Molecular Cloning Technique*. Berger, S.L. & Kimmel, A.R. (eds), pp 180–183, Academic Press. Inc. Press: San Diego, CA, USA.

HOLLSTEIN, M., SIDRANSKY, D., VOGELSTEIN, B. & HARRIST, C.C. (1991). *p53* mutations in human cancers. *Science*, **253**, 49–53.

HSU, I.C., METCALF, R.A., SUN, T., WELSH, J.A., WANG, N.J. & HARRIS, C.C. (1991). Mutational hotspot in the *p53* gene in human hepatocellular carcinoma. *Nature*, **350**, 427–428.

ISAACS, W.B., CARTER, B.S. & EWING, C.M. (1991). Wild-type *p53* suppresses growth of human prostate cancer cells containing mutant *p53* alleles. *Cancer Res.*, **51**, 4716–4720.

JAPANESE HEALTH WELFARE STATISTICS ASSOCIATION (1991). *Health and Welfare Statistics*, **38**, pp 417–418, (In Japanese), Tokyo, Japan.

JAPANESE UROLOGICAL ASSOCIATION AND THE JAPANESE PATHOLOGICAL SOCIETY (1992). General Rule for Clinical and Pathological Studies on Prostatic Cancer. Ed. 2. (In Japanese). pp 40–46, Kanehara Press: Tokyo, Japan.

KAIGHN, M.E., SHANKAR NARAYAN, K., OHNUKI, Y., LECHNER, J.F. & JONES, L.W. (1979). Establishment and characterization of a human prostatic carcinoma cell line (PC-3). *Invest. Urol.*, **17**, 16–23.

KASAI, K., KAMEYA, T., KADOYA, K. & WADA, C. (1991). A pulmonary large cell carcinoma cell line expressing neuroendocrine cell markers and human chorionic gonadotropin-subunit. *Jpn. J. Cancer Res.*, **82**, 12–18.

LANE, D.P. & CRAWFORD, L.V. (1979). T antigen is bound to a host protein in SV40 transformed cells. *Nature*, **278**, 261–263.

LEVINE, A.J., MOMAND, J. & FINLAY, C.A. (1991). The *p53* tumor suppressor gene. *Nature*, **351**, 453–456.

MARSHALL, C.J. (1991). Tumor suppressor genes. *Cell*, **64**, 313–326.

MASHIYAMA, S., MURAKAMI, Y., YOSHIMITO, T., SEKIYA, T. & HAYASHI, K. (1991). Detection of *p53* gene mutations in human brain tumors by single-strand conformation polymorphism analysis of polymerase chain reaction products. *Oncogene*, **6**, 1313–1318.

McCULLOUGH, D.L. (1988). Diagnosis and staging of prostatic cancer. In *Diagnosis and Management of Genitourinary Cancer*. Skinner, D.G. & Lieskovsky, G, (ed.), pp 405–406, W.B. Saunders Co Press: Philadelphia, PA, USA.

MERCER, W.E., AMIN, M., SAUVE, G.J., APPELLA, E., ULLRICH, S.H. & ROMANO, J.W. (1990). Wild type human *p53* is antiproliferative in SV40-transformed hamster cells. *Oncogene*, **5**, 973–980.

NIGRO, J.M., BAKER, S.J., PREISINGER, A.C., JESSUP, J.M., HOSTETTER, R., CLEARY, K., BIGNER, S.H., DAVIDSON, N., BAYLIN, S., DEVILEE, P., GLOVER, T., COLLINS, F.S., WESTON, A., MODALLI, R., HARRIS, C.C. & VOGELSTEIN, B. (1989). Mutations in the *p53* gene occur in diverse human tumour types. *Nature*, **342**, 705–708.

OKAMOTO, A., SAMESHIMA, Y., YOKOYAMA, S., TERAHASHI, Y., SUGIMURA, T., TERADA, M. & YOKOTA, J. (1991). Frequent allelic losses and mutations of the *p53* gene in human ovarian cancer. *Cancer Res.*, **51**, 5171–5176.

ORITA, M., IWAHANA, H., KANAZAWA, H., HAYASHI, K. & SEKIYA, T. (1989). Detection of polymorphism of human DNA by gel electrophoresis as single-strand conformation polymorphisms. *Proc. Natl Acad. Sci. USA*, **86**, 2766–2770.

SILVERBERG, E.S., BORING, C.C. & SQUIRTES, T.S. (1990). Cancers statistics 1990. *CA Cancer J. Clin.*, **40**, 9–26.

STONE, K.R., MICKEY, D.D., WUNDERLI, H., MICKEY, G.H. & PAULSON, D.F. (1978). Isolation of a human prostate carcinoma cell line (DU 145). *Int. J. Cancer*, **21**, 274–281.

THOMPSON, S.J., MELLON, K., CHARLTON, R.G., MARSH, C., ROBINSON, M. & NEAL, D.E. (1992). *p53* and Ki-67 immunoreactivity in human prostate cancer and benign hyperplasia. *Br. J. Urol.*, **69**, 609–613.

THOMPSON, T.C., SOUTHGATE, J., KITCHENER, G. & LAND, H. (1989). Multistage oncogenesis induced by *ras* and *myc* oncogenes in a reconstituted organ. *Cell*, **56**, 917–930.

THOMPSON, T.C. (1990). Growth factors and oncogenes in prostate cancer. *Cancer Cells*, **2**, 345–354.

TSAI, Y.C., NICHOLS, P.W., HITI, A.L., WILLIAMS, Z., SKINNER, D.G. & JONES, P.A. (1990). Allelic loss of chromosome 17p distinguishes high grade from low grade transitional cell carcinoma of the bladder. *Cancer Res.*, **50**, 7081–7083.

VOGELSTEIN, B., FEARON, E.R., HAMILTON, S.R., KERN, S.E., PREISINGER, A.C., LEPPERT, M., NAKAMURA, Y., WHITE, R., SMITH, A.M.M. & BOS, J.L. (1988). Genetic alterations during colorectal-tumor development. *N. Engl. J. Med.*, **318**, 525–532.

WADA, C., AKAHOSHI, T., KAMEYA, T., OHTANI, H. & KASAI, K. *p53* mutation in pulmonary large cell carcinoma with neuroendocrine differentiation: a study using reverse transcriptase polymerase chain reaction (RT-PCR) and direct sequencing. (manuscript submitted).

WALLACE, M.R., MARCHUK, D.A., ANDERSON, L.B., LETCHER, R., ODEH, H.M., SAULINO, A.M., FOUNTAIN, J.W., BRERETON, A., NICHOLSON, J., MITCHELL, A.L., BROWNSTEIN, B.H. & COLLINS, F.S. (1990). Type 1 neurofibromatosis gene: Identification of a large transcript disrupted in three *NF1* patients. *Science*, **249**, 181–186.

WATERHOUSE, J., MUIR, C., SHANMUGARATNAM, K. & POWELL, J. (eds) (1982). Cancer Incidence in Five Countries. Vol. 4, IARC Scientific Publication 42, pp. 896, Lyon: International Agency for Research on Cancer.

WESTON, A., WILLEY, J.C., MODALI, R., SUGIMURA, H., McDOWELL, E.M., RESAU, J., LIGHT, B., HAUGEN, A., MANN, D.L., TRUMP, B.F. & HARRIS, C.C. (1989). Differential DNA sequence deletions from chromosome 3, 11, 13, and 17 in squamous-cell carcinoma, large-cell carcinoma, and adenocarcinoma of the human lung. *Proc. Natl Acad. Sci. USA*, **86**, 5099–5103.

YAMADA, Y., YOSHIDA, T., HAYASHI, K., SEKIYA, T., YOKOTA, J., HIROHASHI, S., NAKATANI, K., NAKANO, H., SUGIMURA, T. & TERADA, M. (1991). *p53* gene mutations in gastric cancer metastases and in gastric cancer cell lines derived from metastases. *Cancer Res.*, **51**, 5800–5805.

YATANI, R., KUSANO, I., TAKANARI, H., NAKANO, H., OKINAKA, T. & KOMADA, S. (1989). International comparison of prostatic carcinoma (In Japanese). In Sugano, H. (ed.), *Recent Topics in Cancer of Several Organs*, pp. 239–245, Tokyo: Shinohara Shuppan.